# Supplementary material for: A case study of percutaneous epidural stimulation to enable motor control in two men after spinal cord injury
Source: Nat Commun. 2023 Apr 12;14:2064. doi: 10.1038/s41467-023-37845-7 (PMC10091329; doi:10.1038/s41467-023-37845-7)
Supplement: Supplementary file 1 — Supplementary Information [file 41467_2023_37845_MOESM1_ESM.docx]

A Case Study of Percutaneous Epidural Stimulation to Enable Motor Control

in Two Men after Spinal Cord Injury

**Supplementary Information**


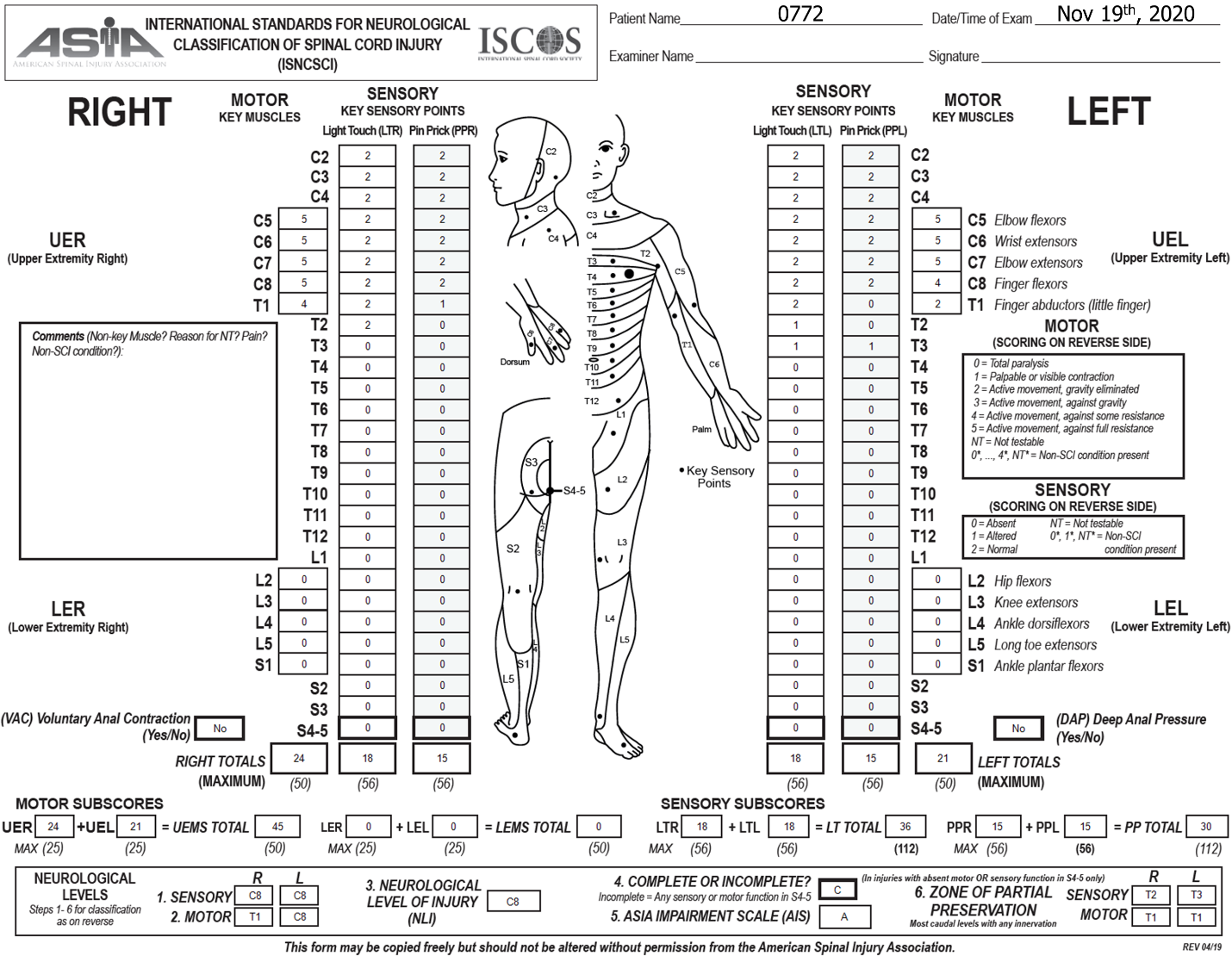


**Supplementary Figure 1.** American Spinal Injury Association Impairment Scale exam sheet for 0772.


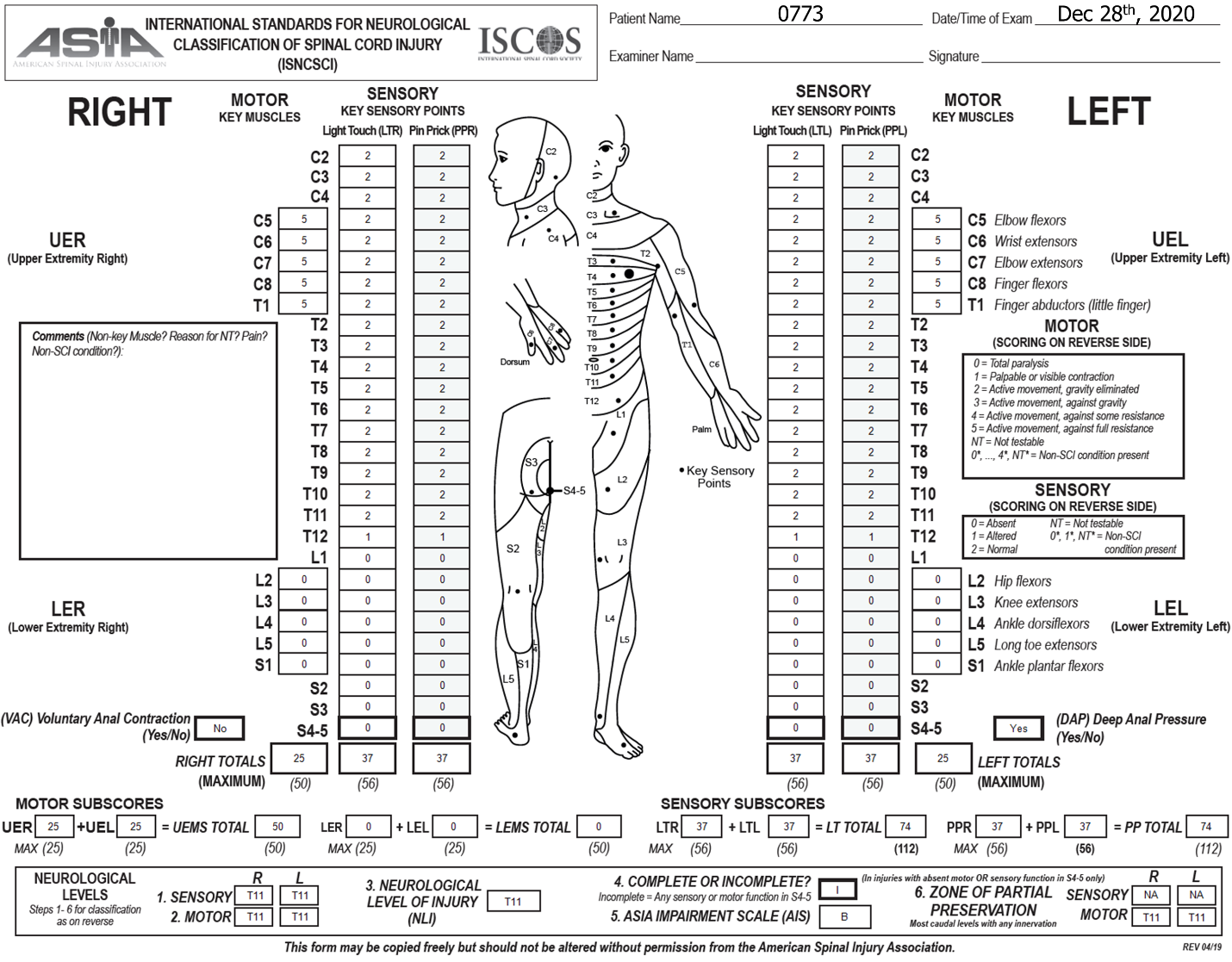


**Supplementary Figure 2.** American Spinal Injury Association Impairment Scale exam sheet for 0773.


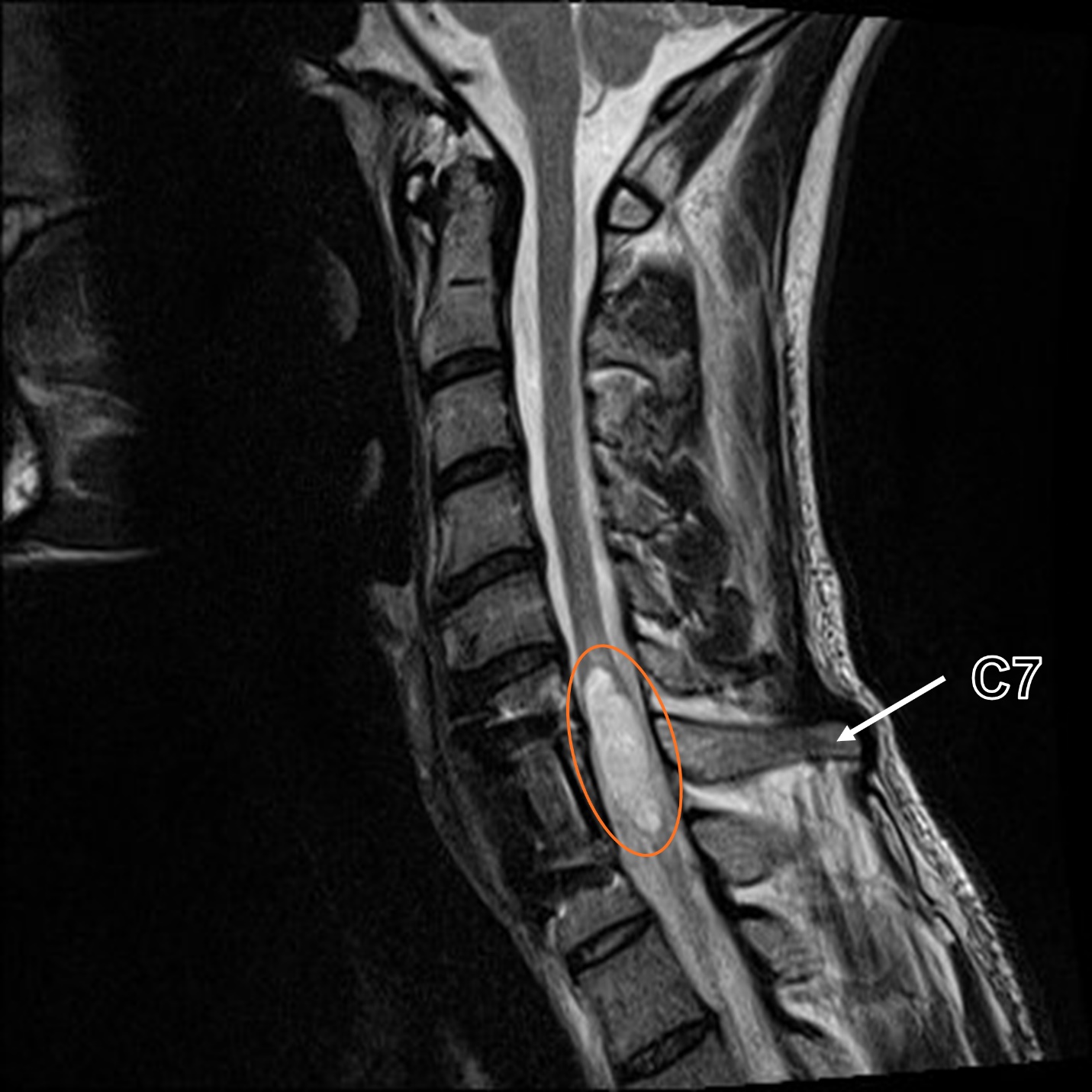


**Supplementary Figure 3.** **MRI for 0772.** Magnetic resonance image (T2 Turbo Spin Echo with long band width; SIEMENS 1.5T) of the cervical spine for 0772. The injury to the cervical cord (marked with the orange oval) spans from approximately the C6 to T1 vertebrae, with an area of approximately 3.25 x 0.9 cm^2^. Magnetic resonance images for 0773 are not available due to spinal hardware.


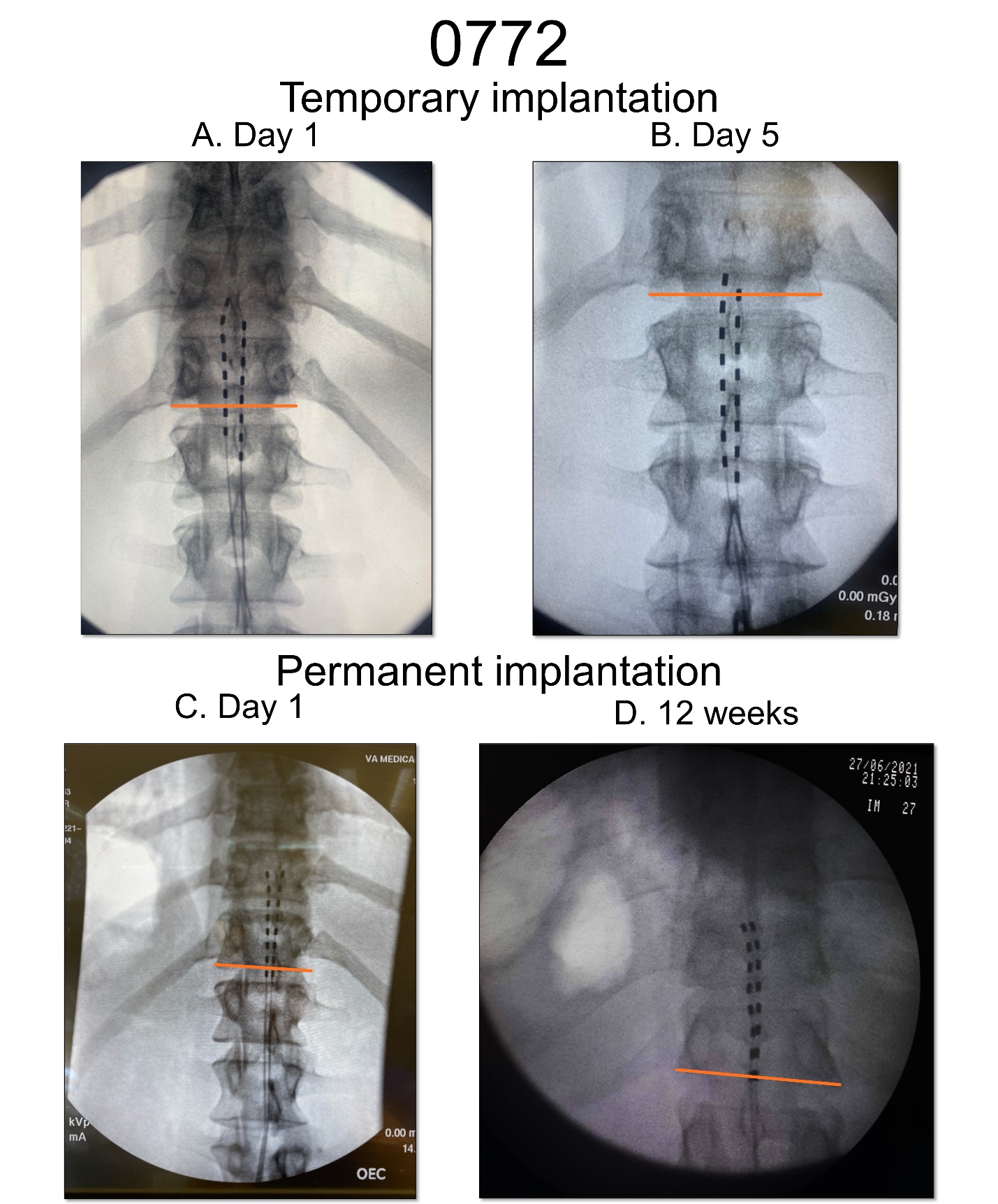


**Supplementary Figure 4.** **Percutaneous leads for 0772.** Images showing position of the leads at different timepoints for temporary (A, B) and permanent (C, D) implantation for 0772. In all images, the distal border of the T12 vertebra is marked with an orange line to allow comparison between images. On Day 1 of temporary implantation (A), the proximal ends of the temporary left and right leads were placed at 0.9 and 1.41 cm, respectively, distal to the proximal rim of T11 in 0772. On the 5^th^ day (B), the left and right leads migrated distally 4.9 and 5.53 cm, respectively, from the proximal rim of T11 vertebra. The spacing between the left and right leads also decreased from 0.67 to 0.38 cm from day 1 to day 5. Temporary implantation was conducted to successfully ensure activation of the lumbosacral segments prior to conducting permanent implantation.

On the day of permanent implantation (C), the leads were placed in approximately the same location and slightly more proximal compared to the temporary. Twelve weeks following permanent implant (D), slight caudal migration of both leads is apparent (< 0.4 cm), though not to the same extent to that which occurred within 5 days with the temporary leads.


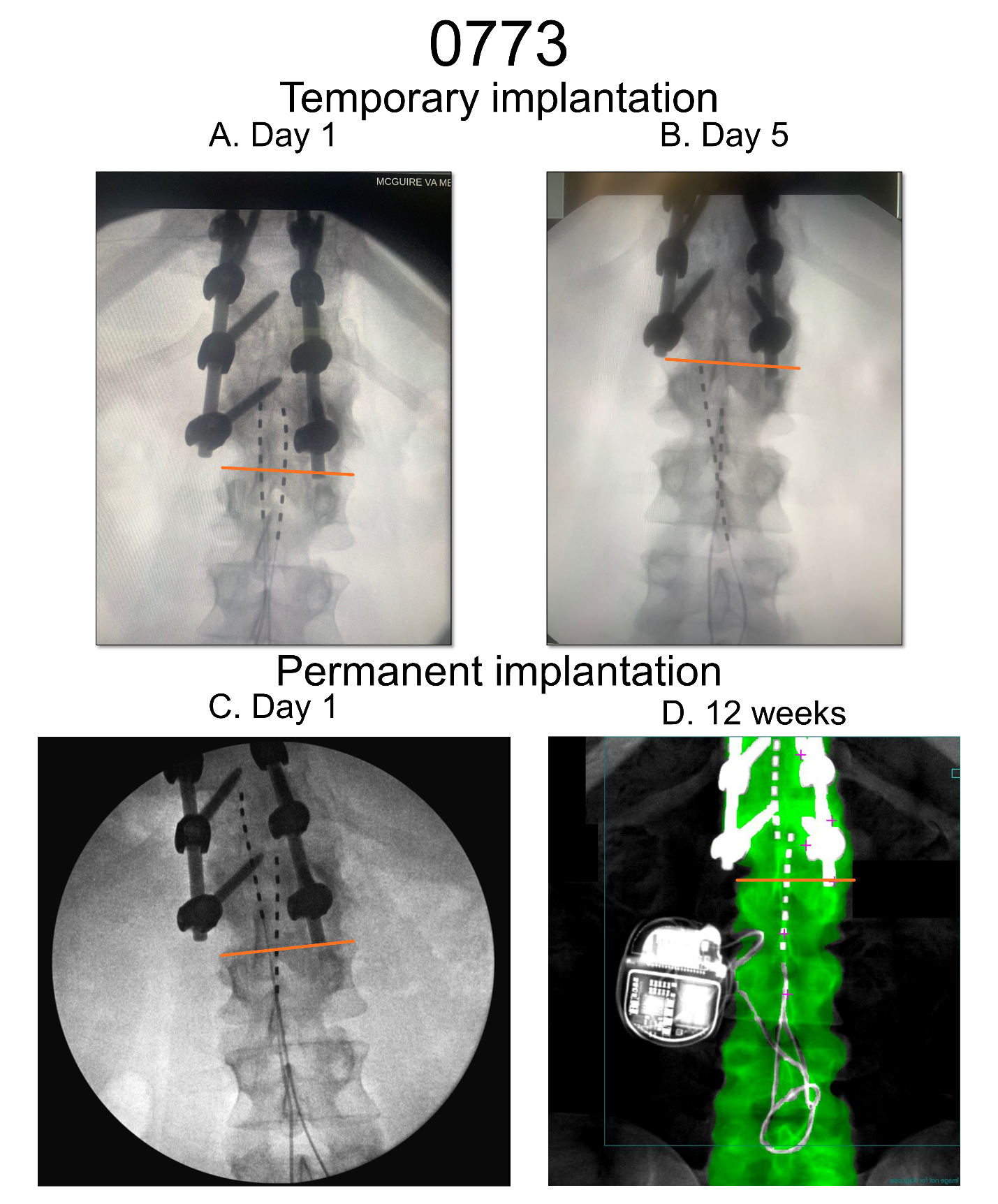


**Supplementary Figure 5. Percutaneous leads for 0773.** Images showing position of the leads at different timepoints for temporary (A, B) and permanent (C, D) implantation for 0773. In all images, the proximal border of the L2 vertebra is marked with an orange line to allow comparison between images. It was possible to thread the left lead up to the T12 vertebra, but excessive fibrous tissue blocked symmetrical threading on the right side. Therefore, the left and right leads were placed 0.50 and 0.92 cm, respectively, distal from the proximal rim of L1 vertebra (A). The spinal fusion extended from T10-L1 vertebrae but did not prevent placement of the leads or appropriate spinal mapping. On Day 5 (B), both leads showed substantial distal migration to below the proximal L2 vertebral border. The crossing over of the leads obfuscated precise measurement to quantify the amount of migration. On the day of permanent implantation (C), the right lead was placed in approximately the same location as the temporary implantation. By clinical judgement of the implanting surgeon, the left lead was threaded to near the proximal border of the T12 vertebrae, to cover more spinal cord segments corresponding to locomotor central pattern generators. 12 weeks from permanent implantation (D), an anterior-posterior spine dual-energy x-ray absorptiometry scan was conducted as part of routine study evaluations. To minimize radiation exposure to the participant, this image was used for the 12-week comparison of lead location. Slight caudal migration of the left lead and caudal migration of the right lead is apparent (~1.6 cm), though not to the same extent to that which occurred within 5 days with the temporary leads.


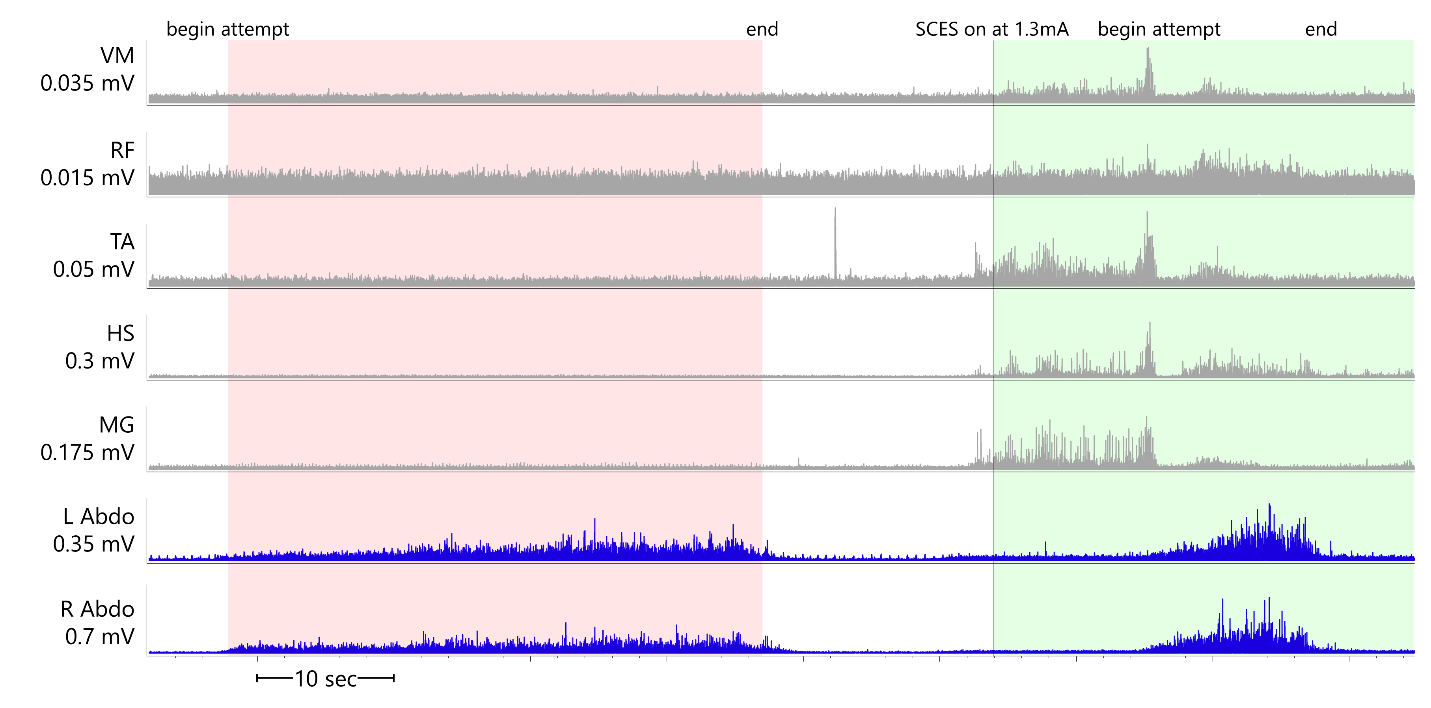


**Supplementary Figure 6. Electromyograms (EMGs) corresponding** All muscles shown are for the right side of the body except the left and right abdominals, which are highlighted in blue. In the red highlighted section, participant 0773 is attempting to flex his right leg without SCES. The abdominal EMGs indicate he is attempting to flex his hip and knee, and while trace movement, no activity is evoked in any lower extremity muscle. In green, SCES is turned on at 1.3 mA, which induced some activity in lower extremity muscles, but not in the abdominals, and no movement was elicited. Once the participant attempted to flex his leg (indicated by the corresponding abdominal activity), corresponding increases in activity in each lower extremity muscle can be seen, notably in the rectus femoris and hamstring, likely giving rise to the enhanced movement. EMGs presented are rectified and bandpass filtered at 10-990 Hz. L, left; R, right; VM, vastus medialis; RF, rectus femoris; TA, tibialis anterior; HS, hamstring; MG, medial gastrocnemius; GM, gluteus medius; Abdo, abdominals; mV, millivolts; mA, milliamps; Hz, hertz; µs, microseconds; sec, seconds.


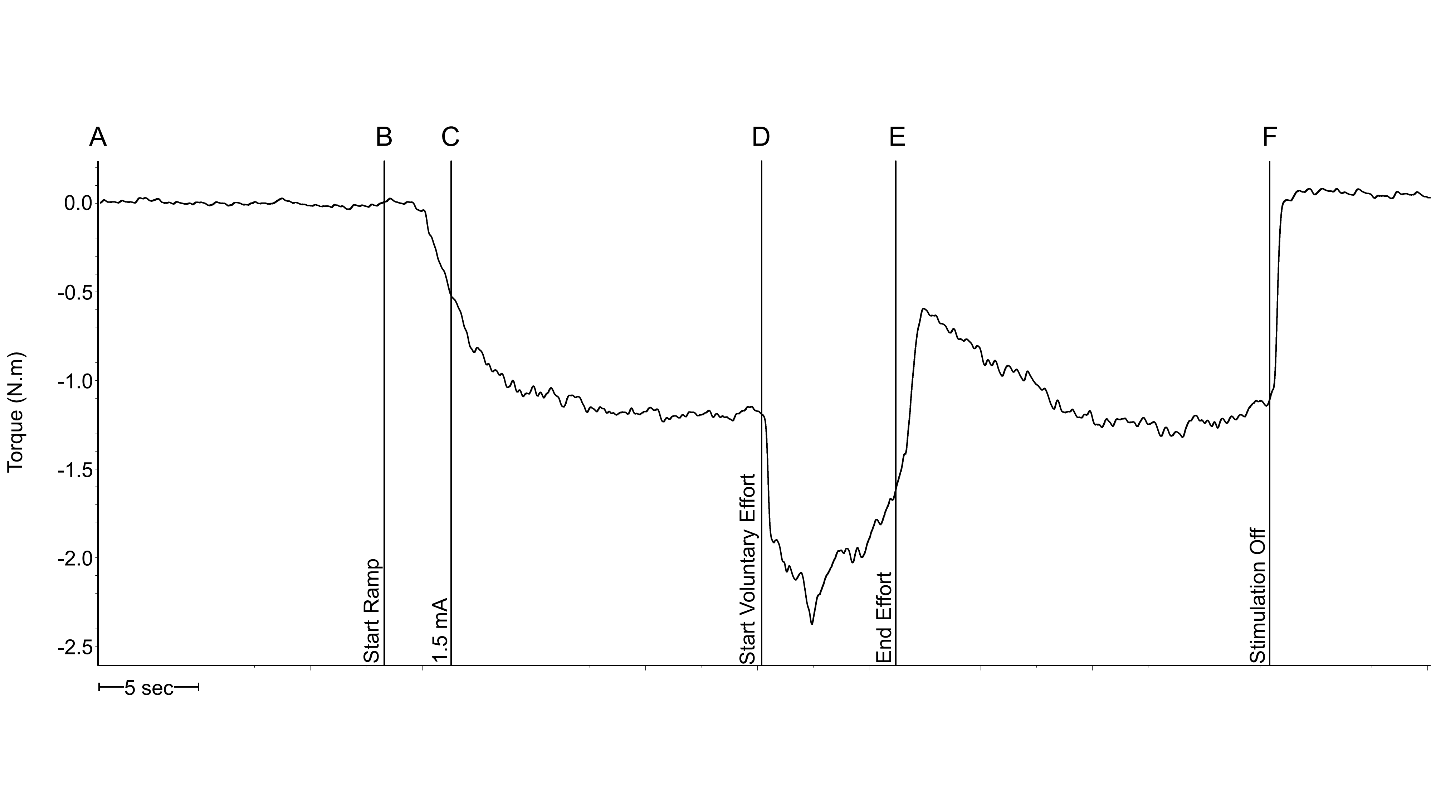


**Supplementary Figure 7.** **Representative trace of induced and voluntarily modulated torque in the presence of SCES**. Sections A-F are periods used to quantify the differences in torque achieved with voluntary effort versus torque induced by SCES, which is fully described in the supplementary methods.

**Supplementary Table 1 – Knee extension torque results of 0772 & 0773 participants and methods for analysis**

|  |  | Participant 0772 | | | | | | |
| --- | --- | --- | --- | --- | --- | --- | --- | --- |
|  |  | **Left leg** | | | | **Right leg** | | |
|  | SCES induced direction | SCES induced TTI (N.m/sec) | Voluntary effort TTI (N.m/sec) | | | SCES induced TTI (N.m/sec) | | Voluntary effort TTI (N.m/sec) |
| 75% MT (20Hz) | Extension (L)/ Extension (R) | 0.03 | 0.2 (↓*) | | | 2.5 | | 1.4(↓E) |
| 100% MT (20Hz) | Flexion (L)/ Extension (R) | 0.02 | 0.1 (↑F) | | | 2.5 | | 1.9 (↓E) |
| 75% MT (34Hz) | Flexion (L)/ Extension (R) | 0.03 | 0.5 (↑F) | | | 2.6 | | 0.9 (↓E) |
| 100% MT (34Hz) | Extension (L)/ Extension ( R) | 0.01 | 0.6 (↓*) | | | 0.7 | | 0.9 (↑E) |
|  |  |  | |  |  | |  | |
|  |  | Participant 0773 | | | | | | |
|  |  | Left leg | | | | Right leg | | |
|  | SCES induced direction | SCES induced TTI (N.m/sec) | Voluntary effort TTI (N.m/sec) | | | SCES induced TTI (N.m/sec) | | Voluntary effort TTI (N.m/sec) |
| 75% MT (20Hz) | Flexion (L)/ Flexion (R) | 0.2 | 2.3 (↑F) | | | 2.2 | | 4.6 (↑F) |
| 100% MT (20Hz) | Flexion (L)/ Flexion (R) | 6.7 | 11.7 (↑F) | | | 2.6 | | 4.7 (↑F) |
| 75% MT (40Hz) | Flexion (L)/ Flexion (R) | 0.2 | 2.0 (↑F) | | | 0.6 | | 3.4 (↑F) |
| 100% MT (40Hz) | Flexion (L)/ Flexion (R) | 2.2 | 3.6 (↑F) | | | 1.0 | | 3.8 (↑F) |

In all cases, when delivered in the seated position, SCES at 75% or 100% of supine knee extension motor threshold (MT) induced torques at the knees.

Torque time integral (TTI) in Newton-meters per second (N.m/sec) at baseline was calculated as maximum volitional TTI attempt without SCES minus background TTI. For 0772 participant, baseline TTI was 0.03 Nm/sec and for 0773 participant, TTI was 0.8 and 0.2 Nm/sec for the right and left legs, respectively. For maximal volitional attempts, each participant was asked to extend (i.e. kick) as hard as possible for approximately 5 seconds.

Torque time integrals (TTI) induced by SCES and TTI during voluntary effort with SCES were provided in Table 1. TTI induced by SCES was calculated as the TTI during SCES on minus background TTI for each participant. TTI during volitional effort with SCES on was calculated as TTI of voluntary enabled torque minus TTI of SCES induced torque.

In all instances, the participant’s supine knee extensor SCES configuration was used, though in some instances, knee flexion was induced in the seated position depending on the stimulation parameters. Participants then attempted to extend their knee, but in some instances, generated a flexion torque instead of extension. For 0773, volitional extension TTI was never enabled. L: left leg and R: right leg.

↓*indicates SCES-induced extension torque converted to a flexion torque upon voluntary effort;

↑F – indicates SCES-induced flexion torque increased further into flexion upon voluntary effort; ↓E – indicates SCES-induced extension torque decreased but remained in the extension direction upon voluntary effort;

↑E – indicates SCES-induced extension torque increased further into extension upon voluntary effort.

**Supplementary Methods**

A Biodex dynamometer (Shirely, NY) was used to examine isometric knee extensor torque and was calibrated according to manufacturer guidelines. After transferring using a ceiling lift, the participant was securely strapped to the test chair with a crossover shoulder harnesses and a belt across the hip joint. The axis of the dynamometer was aligned to the anatomical knee axis and the lever arm was attached 2-3 cm above the lateral malleolus. Once the participant was seated and secured in the seat, measurements were taken, and the knee was fixed at a 90-degree angle while the hips were positioned at 110 degrees. Torque measurements were taken with and without epidural stimulation. As previously mentioned, isometric torque measurements were made using two different stimulation amplitudes and two frequencies (see supplementary Table 1). For each condition, the stimulation parameters were selected, and the stimulation took approximately 2-4 seconds to reach the target amplitude. Torque often continued to increase over the course of a few seconds after the target amplitude was reached; therefore, the stimulation was left on for approximately 15 seconds to ensure the induced torque was stabilized. Upon observing approximately 5 seconds of stable induced torque, participants were asked to extend their knee as forcefully as possible for 3-5 seconds. Following the volitional effort, SCES was left on for up to 15 seconds until the torque returned to induced levels, then was turned off and torque returned to baseline levels.

To quantify the difference in torque achieved with voluntary effort versus torque induced by SCES, the torque time integral (TTI) was calculated because the time of ramping SCES to target amplitudes could not be standardized. This was due to the stimulator being pre-programmed to increase amplitudes at a non-modifiable rate set by the manufacturer. Therefore, the TTI of the segments of the testing was calculated to compare the effects of different conditions of SCES. The TTI of each segment was calculated by dividing the integral of the torque signal by the time (seconds) of the segment of interest. The TTI of the no-SCES baseline (segment A-B, supplementary figure 7) was subtracted from the TTI of the SCES-induced torque (C-D) to establish the TTI induced by SCES. The TTI during the participant’s volitional effort (D-E) was then calculated. Results of the torque testing are presented in Supplementary Table 1.
